# Programmed death-ligand 1 (PD-L1) expression in cervical intraepithelial neoplasia and cervical squamous cell carcinoma of HIV-infected and non-infected patients

**DOI:** 10.1007/s00428-023-03580-z

**Published:** 2023-06-21

**Authors:** Maria José Brito, Pedro Sequeira, Ana Quintas, Iryna Silva, Fernanda Silva, Catarina Martins, Ana Félix

**Affiliations:** 1 Hospital Garcia de Orta, Almada, Portugal; 2 Pathology, Champalimaud Clinical Centre, Champalimaud Foundation, Avenida Brasília, 1400-038 Lisbon, Portugal; 3 Instituto Português de Oncologia de Lisboa Francisco Gentil (IPOLFG), Lisbon, Portugal; 4 NOVA Medical Research, NOVA Medical School/Faculdade de Ciências Médicas, Universidade NOVA de Lisboa, Lisbon, Portugal; 5 CHRC, Comprehensive Health Research Center, Lisbon, Portugal

**Keywords:** Cervix, Human immunodeficiency virus, PD-L1, Squamous cell carcinoma, Squamous intraepithelial lesion

## Abstract

Programmed death-ligand 1 (PD-L1) is overexpressed in cervical carcinoma, hindering tumor destruction. The aim of this study was to assess PD-L1 expression by immunohistochemistry in cervical squamous cell carcinoma (SCC) and squamous intraepithelial lesions (SILs) from human immunodeficiency virus–positive (HIV+) and human immunodeficiency virus-negative (HIV-) patients. A total of 166 SCC and SIL samples of HIV+ and HIV- patients were included and analyzed for PD-L1 expression through tumor proportion score (TPS), and results were stratified in five TPS groups using SP263 antibody and, combined positive score (CPS) using 22C3 antibody. In cohort 1 (SP263 clone), all HIV+ patients were negative for intraepithelial lesion or malignancy (NILM), and low-grade squamous intraepithelial lesions (LSILs) scored < 1; and 87.5% of high-grade squamous intraepithelial lesions (HSILs) adjacent to SCC, 19% of HSILs non-adjacent to SCC, and 69% of SCCs scored ≥ 1 (15.4% scored 5). In HIV- patients, all NILM, LSILs, HSILs adjacent to SCC, and two HSILs non-adjacent to SCC scored < 1. SCC: 88.2% scored ≥ 1 and 5.9% scored 5. In cohort 2 (SP263 and 22C3 clones), 16.7% of HIV+ patients with SCC were positive with both clones, CPS ≥ 1 (22C3) or score 5 (≥ 50%) (SP263), showing no significant differences in positivity between both clones. These results indicate that a relatively low percentage of SCCs (16.7%; both in HIV+ and in HIV- patients) express PD-L1 (TPS ≥ 50% and CPS > 1), which may be due to some samples being archival material, sample characteristics, or use of different methodologies, highlighting the need for standardization of PD-L1 assessment in SCC of the cervix. The fact that PD-L1 is overexpressed in SILs of HIV+ patients suggests potential additional applications for immunotherapy in this disease.

**Supplementary Information:**

The online version contains supplementary material available at 10.1007/s00428-023-03580-z.

## Introduction

The role of immune checkpoint inhibitors has been explored in several malignancies, including uterine cervical cancer [1–3] due to their ability to interfere with the body’s defense mechanisms against cancer, but this role has been scarcely studied in the progression from squamous intraepithelial lesions (SILs) to squamous cell carcinoma (SCC).

Programmed cell death protein-1 (PD-1) and its ligand programmed death-ligand 1 (PD-L1) are coinhibitory regulators that suppress proliferation and cytokine production by CD8^+^ T lymphocytic cells, preventing tumor surveillance and destruction by immune cells [4].

Alterations in the PD-1/PD-L1 axis are implicated in the impairment of local immune response to HPV-associated cervical lesions. Immunohistochemistry (IHC) analysis in cervical intraepithelial neoplasia and cervical cancer revealed no PD-L1 expression in normal cervical epithelium (even when adjacent to cancer), but conversely showed PD-L1 expression in 95% of SILs and 80% of cervical SCCs (both in epithelial squamous and immune cells) [5]. Moreover, in patients with cervical cancer, an association was found between HPV-infected cells, increased number of CD8^+^ T-cells, and increased PD-L1 and PD-1 expression in the setting of progression from normal cervical epithelium to invasive cervical cancer [5–9].

CD8^+^ T lymphocytes are the primary inflammatory cells expressing PD-L1 and are strongly clustered around SILs and nests of invasive squamous cancer cells, suggesting that PD-L1 is upregulated in carcinoma and cervical cancer microenvironment [5].

Besides inhibiting T-cell proliferation and cytokine production, PD-L1 also promotes T-cell activation, although the explanation for this paradox remains elusive [10–13].

In chronic human immunodeficiency virus (HIV) infection, HIV-1 virions induce high levels of PD-L1 expression and resulting in an inhibitory effect on immune cells, like T-cells and neutrophils [14]. In addition, PD-L1 is expressed in CD4+Foxp3+ regulatory T-cells (T regs), which play an important role in promoting immunosuppression [15].

This may explain why the local immune response is not effective in preventing the development of cervical SCC in patients with HIV-positive (HIV+) infection and HPV-associated cervical epithelial lesions.

The rates of IHC detection of PD-L1 positivity in cervical specimens from patients not infected with HIV vary between 32% and 80% [5, 16, 17]. This high variation has been attributed to several factors, such as tissue sample characteristics (SILs vs. invasive cancer), cancer stage (early vs. metastatic), history of chemotherapy treatment and combination antiretroviral therapy (cART)–treated patients exhibiting a lower prevalence of PD-L1 immunopositivity [18]. In addition, the antibodies, quantification methods, and thresholds employed have also been acknowledged as important factors in PD-L1 tissue expression variation and it is unanimous among the scientific community the need for PD-L1 histopathological testing validation and standardization [19].

The primary aim of this study was to assess PD-L1 expression in cervical lesions from HIV+ patients treated in Portugal. SIL and SCC specimens were used to investigate PD-L1 expression in the context of tumor progression from the initial carcinogenesis steps to invasive carcinoma in HIV+ versus HIV- patients. The study’s secondary aim was to compare two different approaches to PD-L1 IHC assessment in invasive carcinoma using different antibodies and scoring systems.

## Material and methods

This was an observational, cross-sectional study of HIV+ and HIV- patients with SILs or SCC followed at two health institutions in Portugal: Hospital Garcia de Orta, in Almada, and Instituto Português de Oncologia de Lisboa Francisco Gentil, in Lisbon.

Two patient cohorts were included. To address the primary study objective, a cohort of patients with SILs and SCC was included (cohort 1), and the SP263 antibody (Roche/Ventana) was used for IHC analysis. To address the secondary study objective, a second cohort of only SCC patients was included (cohort 2), and two antibodies were used for IHC analysis: SP263 (Roche/Ventana) and 22C3 (Dako). Samples from HIV+ and HIV- patients were included in both cohorts.

Cohort 1 comprised 140 cervical specimens of SILs and invasive carcinoma, of which 70 were from HIV+ (1 hysterectomy and 69 biopsies) and 70 from HIV- (61 biopsies and nine cone biopsies) patients. These specimens were assessed by IHC using the anti-PD-L1 antibody SP263. HIV+ samples comprised 13 invasive SCCs, eight high-grade squamous intraepithelial lesions (HSILs) adjacent to SCC, 21 HSILs non-adjacent to SCC, 20 low-grade squamous intraepithelial lesions (LSILs), and 20 negative for intraepithelial lesion or malignancy (NILM) samples. HIV- samples included 17 invasive SCCs, 9 HSILs adjacent to SCC, 20 HSILs non-adjacent to SCC, 20 LSILs, and 20 NILM. All slides were double-checked to confirm the diagnosis.

Cohort 2 comprised 35 SCC specimens, of which 18 were from HIV+ and 17 from HIV- patients. Half of invasive SCC cases (*n* = 9) were also included in the first cohort. IHC assessment was performed using the anti-PD-L1 antibody 22C3. HIV+ samples comprised 15 biopsies, one hysterectomy, and two cone resections, and HIV- samples comprised one biopsy and 16 cone resections. All slides were double-checked to confirm the diagnosis.

### Immunohistochemistry methodology

IHC was performed on 4-μm-thick sections. All slides were stained on the BenchMark ULTRA IHC/ISH 136 Automatic staining platform (Ventana Medical Systems) using appropriate positive and negative controls.

PD-L1 qualitative immunohistochemical assay was conducted using the anti-human PD-L1 monoclonal antibody SP263 (Roche/Ventana) following the manufacturer’s instructions: 16-min incubation at 36 °C in combination with Ventana CC1 (64 min). The anti-human PD-L1 monoclonal antibody 22C3 (Dako) was used at a 1:40 dilution for 28 min with pre-treatment ULTRA CC1 for 48 min (catalogue number M3653, Agilent Dako). Antigen detection was performed with the OptiView DAB IHC Detection Kit (Ventana Medical Systems) using diaminobenzidine as chromogen for detecting expression. Tissue sections were counterstained with Mayer’s hematoxylin.

### Immunostaining assessment

SP263 expression was evaluated by immunostaining in neoplastic epithelial cells according to VENTANA PD-L1 (SP263) Assay Staining Guide after choosing one histological section with high staining for each type of lesion/area. Membrane staining in epithelial cells was considered positive if a brown color of strong or moderate intensity was observed in basolateral or all cell membrane, and the percentage of stained tumor cells in total tumor cells (or tumor proportion score, TPS) in the chosen area was estimated [20]. Based on cut-offs established in previous studies, results were stratified in five TPS groups according to the percentage of positive tumor cells: 0 (0%), 1 (≥ 1% and < 5%), 2 (≥ 5% and < 10%), 3 (≥ 10% and < 20%), 4 (≥ 20% and < 50%), and 5 (≥ 50%) [21].

22C3 expression was evaluated by immunostaining using the combined positive score (CPS) as a measure of expression, as reported in the KEYNOTE⁠-⁠158 trial [22]. This scoring method was approved by the Food and Drug Administration (FDA) as a companion test for determining eligibility for treatment with pembrolizumab in advanced cervical cancer. CPS evaluates the number of PD⁠-⁠L1-staining cells (tumor cells, lymphocytes, macrophages) relative to all viable tumor cells using the formula of total PD-L1 positive cells / total tumor cells (positive + negative) × 100. The maximum CPS score is defined as CPS 100. Any perceptible and convincing partial or complete linear membrane staining of viable tumor cells recognized as distinct from cytoplasmic staining at ×20 magnification is considered positive and included in the scoring method. A minimum of 100 viable tumor cells in the stained slide is required to consider the specimen adequate for PD-L1 assessment. Several areas of the slide (at least five) with tumor and immune cells should be evaluated. Likewise, any membrane and/or cytoplasmic staining of mononuclear inflammatory cells within tumor nests and/or adjacent supporting stroma is considered positive and included in the CPS numerator. Neutrophils, eosinophils, plasma cells, and immune cells associated with the tumor, in situ carcinoma, normal structures, or ulcers are excluded from the CPS score.

Statistical analysis was performed using GraphPad Prism Software (version 9 for Windows, GraphPad Software LLC).

## Results

### Cohort 1

A total of 168 different specimen areas were assessed with the SP263 anti-PD-L1 antibody using the previously defined five TPS groups according to the percentage of positive tumor cells (Table 1).

**Table 1:** Cohort 1: PD-L1 scores in SILs and SCC of the uterine cervix using SP263 antibody

Anti-PD-L1 (SP263)	Diagnosis									
	NILM		LSIL		Adjacent HSIL *		Non-adjacent HSIL		Invasive SCC #	
	HIV+	HIV-	HIV+	HIV-	HIV+	HIV-	HIV+	HIV-	HIV+	HIV-
Score 0	20	20	20	20	1	9	17	20	4	1
Score 1	0	0	0	0	4	0	4	0	2	4
Score 2	0	0	0	0	2	0	0	0	1	1
Score 3	0	0	0	0	0	0	0	0	1	2
Score 4	0	0	0	0	1	0	0	0	3	6
Score 5	0	0	0	0	0	0	0	0	2	1
Total positive/total no. areas (%)	0/20 (0%)	0/20 (0%)	0/20 (0%)	0/20 (0%)	7/8 (87.5%)	0/9 (0%)	4/21 (19.0%)	0/20 (0%)	9/13 (69.2%)	14/17 (82.4%)
Score 5/total no. areas (%)	0/20 (0%)	0/20 (0%)	0/20 (0%)	0/20 (0%)	0/8 (0%)	0/9 (0%)	0/21 (0%)	0/20 (0%)	2/13 (15.4%)	1/17 (5.9%)

*p = 0.0004 (total positive/total cases); #*p* = 0.5645 (total score 5/total cases)

*HIV*, human immunodeficiency virus; *NILM*, negative for intraepithelial lesion or malignancy; *LSIL*, low-grade squamous intraepithelial lesion; *HSIL*, high-grade squamous intraepithelial lesion; *SCC*, squamous cell carcinoma; *PD-L1*, *programmed death-ligand 1*; *no.*, number

**Fig. 1:**
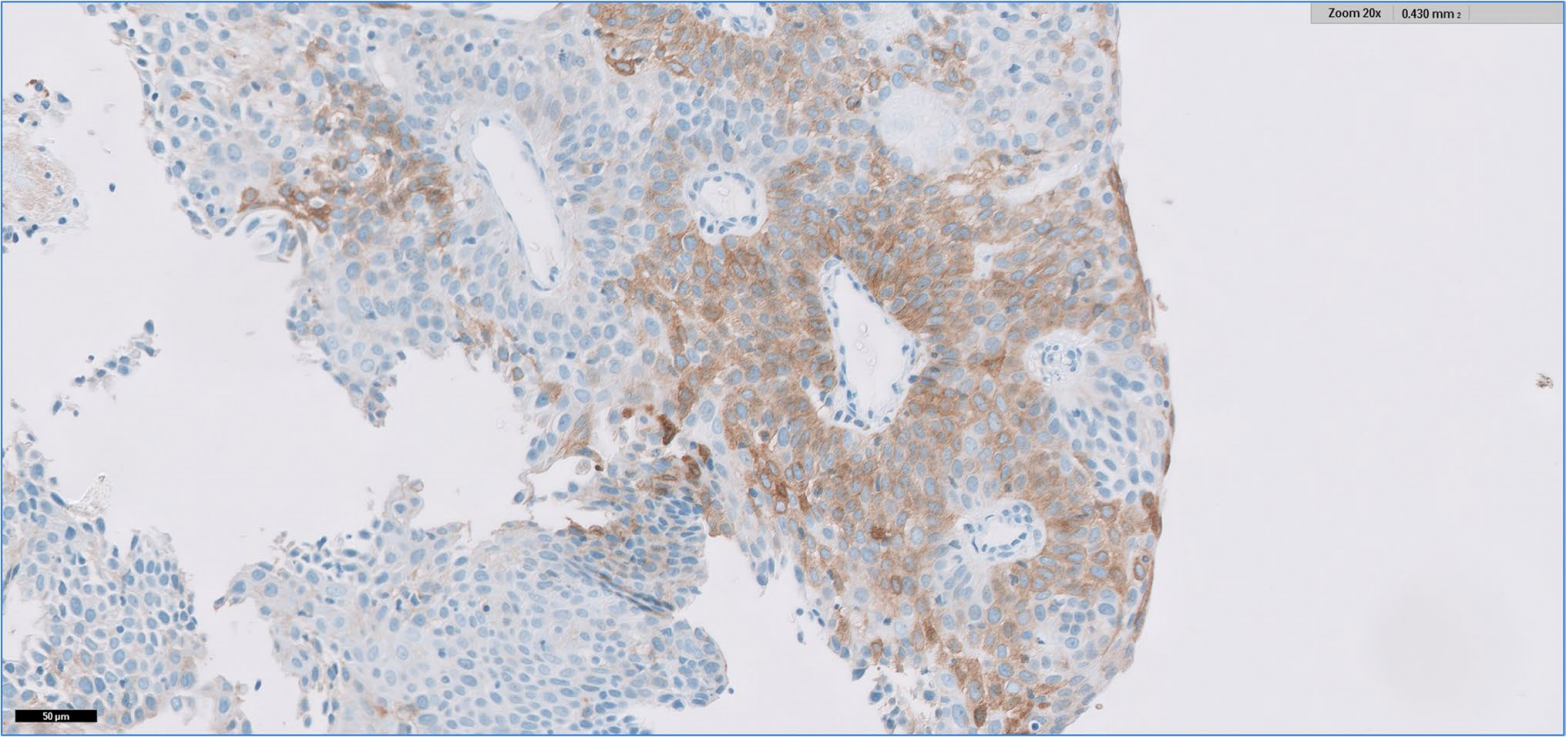
HSIL adjacent to invasive carcinoma (SP263 Roche/Ventana antibody)

**Fig. 2:**
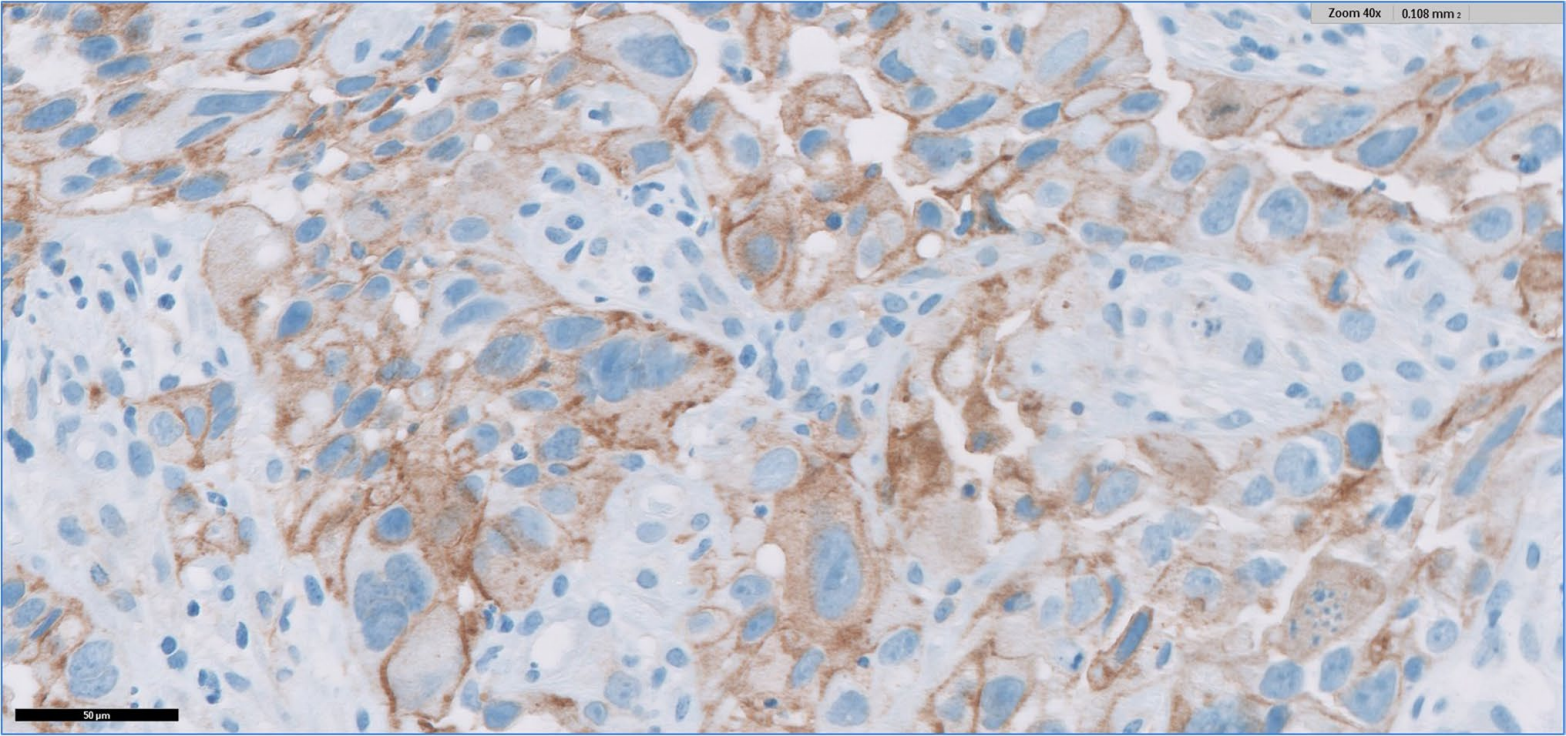
SCC (SP263 Roche/Ventana antibody)

In HIV+ patients, 20 NILMs and 20 LSILs from 20 different cases had a TPS of 0. Among the 8 HSILs adjacent to invasive carcinoma from 8 different samples, one had a TPS of 0, four had a TPS of 1, and two had a TPS of 2 (7/8 [87.5%] areas scored ≥ 1), and among 21 HSILs non-adjacent to carcinoma from 21 different samples, 17 had a TPS of 0 and four had a TPS of 1 (4/21 [19%] areas scored ≥ 1) (Fig. 1). In SCC, four areas scored 0 and 9/13 areas (69%) scored ≥ 1, with the following distribution: two had a TPS of 1, one had a TPS of 2, one had a TPS of 3, three had a TPS of 4, and two (15.4%) had a TPS of 5 (Fig. 2). Cases with TPS of 4 and 5 corresponded to 38.5% of the total number of cases. In HIV- patients, all NILMs and LSILs from 40 different cases had a TPS of 0, as well as 9 HSILs adjacent to invasive carcinoma and 20 HSILs non-adjacent to carcinoma from 20 different cases.

In the 17 HIV- patients with invasive SCC, three had a TPS of 0 and the remaining 14 had a TPS ≥ 1. Among the latter, three had a TPS of 1, one had a TPS of 2, one had a TPS of 3, eight had a TPS of 4, and one (6%) had a TPS of 5. Cases with a TPS of 4 and 5 accounted for 41% of the total number of SCCs.

The number of SCC cases with a TPS of 5 was higher in the HIV+ (2/13 cases [15.4%]; Fig. 2) compared to the HIV- (1/17 cases [5.9%]) subgroup, although without statistical significance (*p* = 0.5645; Table 1).

Regarding HSILs adjacent to invasive SCC (Fig. 1), a significant difference was found in PD-L1 positivity when comparing HIV+ to HIV- patients, with HIV patients showing higher levels (*p* = 0.0004; Table 1). No significant differences were found in PD-L1 positivity (negative vs. positive) between the two groups for all the other types of lesions assessed.

In LSILs and NILMs, no area displayed PD-L1 positivity with the SP263 antibody (Table 1).

### Cohort 2

The results of cohort 2 are depicted in Table 2. In HIV+ patients, SP263 antibody analysis of PD-L1 expression was positive in 66.7% (12/18) of SCCs (TPS ≥ 1), of which five cases had TPS = 0, one had TPS = 1, two had TPS = 2, six had TPS = 4, and two had TPS = 5 (11.1%). SCC cases with TPS of 4 or 5 accounted for 44.4% of the total number of cases.

**Table 2:** PD-L1 scores in invasive SCC from HIV+ and HIV- patients using 22C3 and SP263 antibodies

Case no.	SCC cases in HIV+		Case no.	SCC cases in HIV-	
	PD-L1 (22C3) CPS groups	PD-L1 (SP263) TPS groups		PD-L1 (22C3) CPS groups	PD-L1 (SP263) TPS groups
1	0	4	I	< 1	4
2	0	5	II	0	2
3	9	4	III	0	4
4	0	4	IV	0	1
5	0	4	V	0	4
6	0	0	VI	<1	5
7	0	0	VII	0	3
8	0	5	VIII	0	1
9	0	0	IX	0	4
10	1	4	X	0	0
11	0	0	XI	0	1
12	0	2	XII	0	0
13	0	0	XIII	<1	4
14	0	0	XIV(*)	0	4
15	6	5	XV	0	4
16	0	2	XVI	0	4
17	0	4	XVII	0	0
18	0	1	XVIII		

CPS groups: CPS < 1 (0); CPS ≥ 1 (1); SP263 scoring groups: 0 (0%), 1 (≥ 1% and < 5%), 2 (≥ 5% and < 10%), 3 (≥ 10% and < 20%), 4 (≥ 20% and < 20%), and 5 (≥ 50%); *biopsy

*HIV*, human immunodeficiency virus; *SCC*, squamous cell carcinoma; *PD-L1*, *programmed death-ligand 1; CPS,* combined positive score

Among the 17 HIV- patients with SCC, only two had a TPS of 0, with all the other cases (88.2%) scoring ≥ 1: three had TPS = 1, one had TPS = 2, one had TPS = 3, eight had TPS = 4, and one (5.9%) had TPS = 5. Cases with TPS of 4 or 5 accounted for 52.9% of the total invasive SCC cases.

**Fig. 3:**
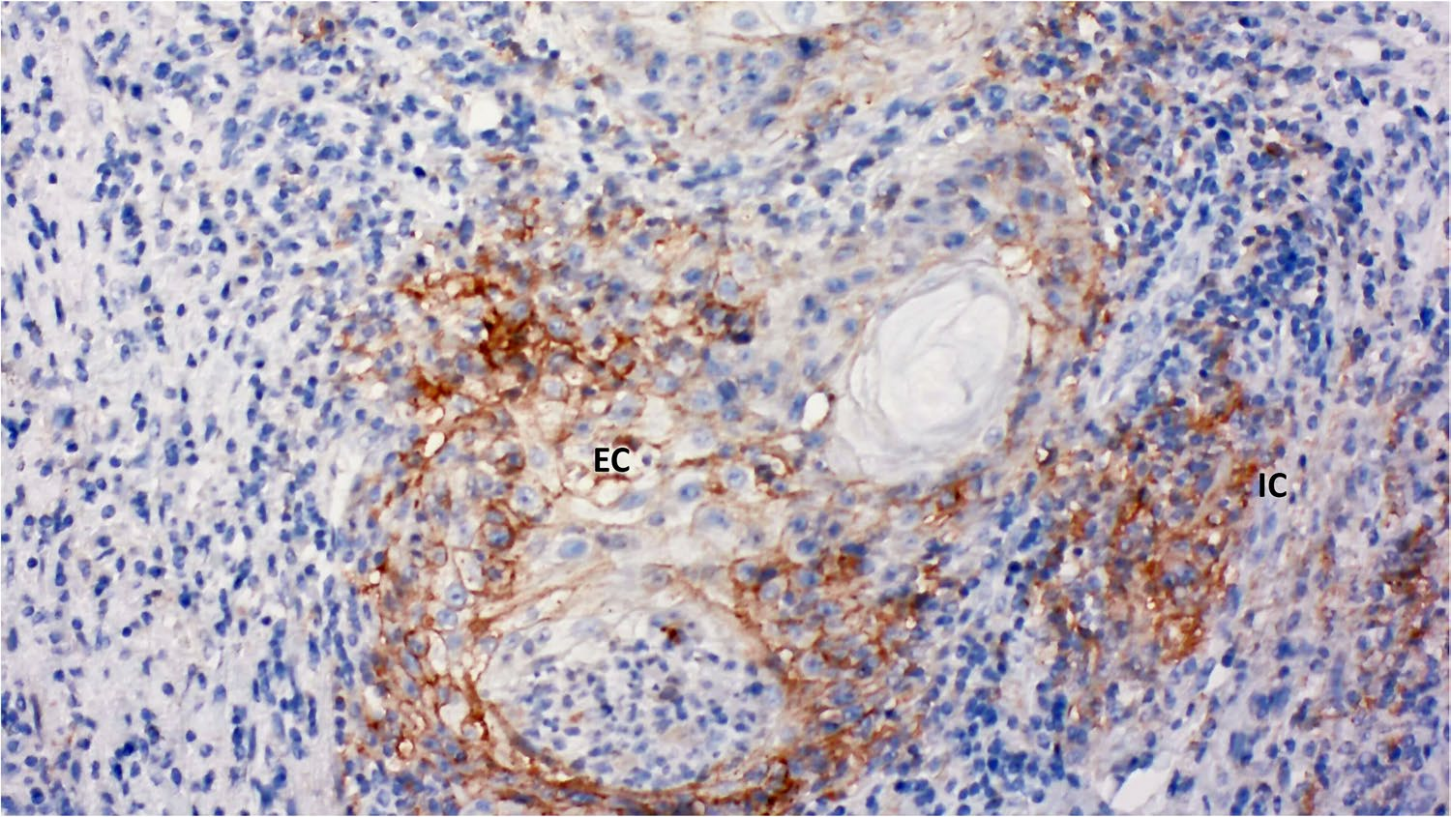
SCC (22C3 Dako atibody)

Figure 3 shows the analysis with the 22C3 antibody. In HIV+ patients, PD-L1 expression was positive (CPS ≥ 1) in three out of 18 (16.6%) invasive SCC cases, with a CPS of 9, 6, and 1, respectively. In contrast, all invasive SCCs from HIV- patients (*n* = 17) were PD-L1 negative (CPS < 1). The three HIV+ cases that were positive for PD-L1 expression in the 22C3 antibody analysis (CPS ≥ 1) were also positive in the SP263 antibody analysis, showing high TPS (2 cases with TPS = 4, and 1 case with TPS = 5; Table 1).

In the SP263 antibody analysis, although more SCC cases were PD-L1-positive in the HIV- group (69.2% HIV+; 82.4% HIV-), no significant differences were found in PD-L1 positivity (TPS ≥ 1) between HIV+ and HIV- patients. Conversely, different results were found in the 22C3 antibody analysis, with a higher number of PD-L1-positive cases in the HIV+ SCC population. Positive CPS ≥ 1 (22C3) was found in 16.7% of SCC cases in HIV+ patients, compared to a null score in 0% of HIV- SCC cases (Table 1). However, when considering the highest PD-L1 threshold (TPS 5 [≥ 50%] for SP263 antibody), more cases were found in HIV+ compared to HIV- patients (15.4% vs. 5.9%).

Table 3 depicts the differences in PD-L1 expression in SCC of HIV+ and HIV- patients with 22C3 and SP263 antibodies. Using the 22C3 antibody, CPS ≥ 1 was found in three cases (16.7%) of SCC from HIV+ patients and none (0%) of SCC from HIV- patients, while CPS < 1 was found in 15 cases (83.3%) of SCC from HIV+ patients and 17 cases (100%) of SCC from HIV- patients. Using the SP263 antibody, TPS ≥ 1 was found in 12 cases (66.7%) of SCC from HIV+ patients and in 15 cases (88.2%) of SCC from HIV-patients, while TPS < 1 was found in 6 cases (33.3%) of SCC from HIV+ patients and in two cases (11.8%) of SCC from HIV- patients.

**Table 3:** Cohort 2: PD-L1 expression in invasive SCC from HIV+ and HIV- patients using 22C3 (DAKO) and SP263 antibodies

	PD-L1 antibody			
	22C3		SP263	
	CPS ≥ 1	CPS < 1	TPS ≥ score 1	TPS < score 1
SCC HIV+	3 (16.7%)	15 (83.3%)	12 (66.7%)	6 (33.3%)
SCC HIV-	0 (0%)	17 (100%)	15 (88.2%)	2 (11.8 %)

*HIV*, human immunodeficiency virus; *SCC*, squamous cell carcinoma; *PD-L1*, *programmed death-ligand 1; CPS,* combined positive score; *TPS*, tumor proportion score

Considering only cases with CPS ≥ 1 or TPS 5 (≥ 50% positive tumor cells), 16.7% (3/18) of cases in the HIV+ group had CPS ≥ 1, and 16.7% (3/18) had TPS 5, showing a 100% agreement between both antibody analyses. In the HIV- group, 5.9% of cases had a TPS of 5, with no patients showing CPS ≥ 1, representing a slightly lower agreement of 94.1% between both analyses.

## Discussion

Blockage of the immune response to cancer cells can be bypassed by drugs targeting this immune receptor [2, 17]. Strategies blocking the PD-L1 pathway have the potential to restore the antitumor activity of T-cells and thus be useful to treat malignant neoplasms [23].

The prognostic value of PD-L1 expression in patients diagnosed with invasive cervical carcinoma has been documented [24]. In cervical cancer, TGCA amplifications in CD274 (PD-L1) and PDCD1LG2 (PD-L2) immune targets were found to be associated with this type of cancer [25], and PD-L1 amplification and polysomy have been shown to represent valid prognostic biomarkers in the treatment with anti-PD-L1s [26].

To our knowledge, this is the first study evaluating PD-L1 expression in SCC and SILs from HIV+ patients in a Western population. Considering the TPS threshold of ≥ 50% for SP263 and CPS > 1 for 22C3, a similar percentage of PD-L1 positivity was found in HIV+ patients with both antibodies (16.7%), reaching a 100% positivity concordance between both. This percentage is however lower compared to other HIV+ series that have used the SP263 antibody: 31% positivity in HIV+ locally advanced *cervical cancer* (with a TPS ⩾ 25% threshold for positivity), and 60% positivity when considering the mean percentage of positive tumor cells [27].

A generally higher PD-L1 expression was found in HIV- patients with cervical carcinoma in this study compared to the study by Feng et al. (2018), which investigated 219 SCC patients using the CD274 antibody [16]. The authors reported a PD-L1 positivity of 32.4% (71/219) in cervical SCC, lower than the 82.4% observed in the present study with the SP263 antibody (TPS ≥ 1) in HIV- patients. However, when the 3+ staining threshold (which refers as positive to cases with strong membranous staining) was considered, Feng and colleagues found a considerably higher positivity rate (12.3%; 27/219) than the one found in the present study in HIV- patients: 6% with SP263 (TPS 5) and 0% with 2C23 [16].

The study by Heren et al. in 156 SCCs and 49 adenocarcinomas of the cervix using the E1L3N clone reported positive PD-L1 in 54% of SCC samples [28]. Positivity was assumed when ≥ 5% of tumor cells were positive for PD-L1 and results similar to ours were achieved in the HIV- population. Interestingly, they also showed that diffuse PD-L1 expression was a risk factor for poor disease-free and disease-specific survival outcomes compared to marginal PD-L1 expression on the interface between tumor and stroma, raising awareness of the importance of tumor microenvironment and suggesting that PD-L1 heterogeneity in tissue may at least partly explain the different PD-L1 positivity results reported in the literature.

The therapeutic inhibition of PD-L1 has been proposed in several studies, and its efficacy demonstrated in clinical trials of patients with recurrent or metastatic cervical cancer (KEYNOTE-028, KEYNOTE-158, CheckMate-358) [22, 29, 30]. The FDA approval of the PD-L1 inhibitor pembrolizumab is conditional on the assessment of PD-L1 expression in cancer tissues with a specific companion assay [2]. The use of companion assays for the assessment of PD-L1 expression is a relevant issue, due to the lack of standardization among assays.

Differences in PD-L1 positivity in different studies may be partly attributed to technical conditions (like the age of the paraffin block), to the number of cases included, or even to the percentage of patients under cART and/or different cART durations.

The variability of immunostaining techniques used by different laboratories in these procedures introduces a great level of uncertainty among pathologists [31], with PD-1/PD-L1 status considered an unreliable biomarker to predict patient response in clinical trials of SCC of the uterine cervix [32] and anal canal [33], and reinforces the need for assay standardization. Different strategies have been proposed for assay harmonization, including the use of calibrators [34, 35].

Thirty-five invasive SCC samples (18 HIV+ and 17 HIV-) were assessed in this study using the SP263 and 22C3 antibodies, in line with previous reports using SP263 in gynecologic cancers [18, 36] and 22C3 according to the FDA-approved companion assay for pembrolizumab [37], and considering the TPS threshold of ≥ 50% for SP263 and CPS > 1 for 22C3. A similar percentage of PD-L1 positivity was found in HIV+ patients with the two antibodies (16.7%), reaching a 100% positivity concordance between both.

PD-L1 positivity in cervical SCC was found to vary according to the antibody used, although the differences in the results obtained with SP263 and 22C3 antibodies were not significant (Table 3). Interestingly, Mills’ group validated the PD-L1 IHC assay using the SP263 antibody on Ventana BenchMark ULTRA against the 22C3 antibody and reported a 97% positivity concordance between both at a cut-off of 50% staining for SP263 and CPS ≥ 1 staining for 22C3 [27].

The value of PD-L1 expression has been explored in the HIV population with SCC in several tumor sites. Using the ab153991 antibody, Govindarajan et al. reported a 56% PD-L1 positivity in 41 SCC samples from the anal canal of HIV- patients, similar to what has been reported in cervical carcinoma [38]. Very interesting results were presented by Bushara and colleagues [39], who reviewed data of the high incidence of anal SCC in HIV+ patients (even under cART) and concluded that chronic inflammation due to HIV infection may contribute to increased PD-L1 expression and CD8^+^ T lymphocyte exhaustion, impairing the cytotoxic antitumor response as previously reported by our group [40]. In contrast to these findings, another study in HIV+ and HIV- patients diagnosed with SCC of the anal canal showed that HIV status was not correlated with PD-L1 expression or with the degree or composition of immune cell infiltration and found a significant increase in IL-18 expression in HIV-associated anal SCC [41].

Regarding premalignant lesions, higher PD-L1 positivity rates were found in this study in HIV+ patients with SCC versus SILs and with HSILs adjacent to invasive carcinoma versus HSILs non-adjacent to invasive carcinoma, in contrast with the negative PD-L1 scores found in HSILs (adjacent or non-adjacent to SCC) in the HIV- group. As far as the authors are aware, this result has not been reported in cervical lesions from HIV+ patients to date.

PD-L1 expression in cervical and anal SILs was investigated in three and two studies in the literature, respectively, with results showing a predominantly negative expression in normal epithelium and low to high expression in SILs [5, 9, 42–44]. However, most of these studies were conducted in the HIV- population. In the HIV+ population, only two studies have investigated PD-L1 expression in anal SILs [39, 41]. In the study by Bushara et al., HIV replication in immune cells was shown to increase the secretion of the tat protein that is integrated into epithelial cells of the HPV-infected anal canal, facilitating transcription of the HPV E6 and E7 oncoproteins and being responsible for the higher incidence of SILs in the anal canal of HIV+ patients, as observed in the cervix [39]. In the study by Yanik et al., PD-L1 expression was found to correlate with increased immune cell infiltration and CD8^+^ T-cell density in the immune microenvironment of the anal canal [41]. This finding is in line with previous observations from our group in cervical lesions of HIV+ patients showing that these patients present higher epithelial and stromal CD8^+^ T-cell scores in all cervical epithelial lesion groups (LSIL, HSIL, and SCC) compared to HIV- counterparts [40].

According to one study, PD-1-positive lymphocytes were more frequently observed in HSILs compared to LSILs in anal SCC of HIV+ patients, indicating that these alterations may be relevant for tumor progression and invasion [41]. Interestingly, a complementary observation was done in anal SILs of HIV- patients, suggesting that PD-1 and lymphocytic CD8^+^ T-cells are more commonly observed in high-grade lesions [42].

A clinical trial of the immune checkpoint inhibitor nivolumab in 37 patients with metastatic SCC of the anal canal showed the efficacy of this agent as monotherapy in these patients [43].

Findings of this study regarding PD-L1 expression in HSILs, especially in HIV+ patients, could be further explored, for instance regarding its use as a risk biomarker. As proposed by Brun et al., these lesions may be targeted by different types of therapeutic interventions interfering with checkpoint inhibitors, such as immunotherapy (e.g., peptide/protein-based vaccines, nucleic acid-based vaccines [DNA], and live vector-based vaccines [bacterial or viral]), just before lesion development [42].

Although the low number of cases included in this study is a limitation, the large number of positive PD-L1 cases identified with both antibodies in SCC of HIV+ patients sustain that HIV immunosuppression may have an impact in regulating checkpoint inhibitors, particularly since all patients were under cART. And, the fact that PD-L1 overexpression is observed in intraepithelial squamous cervical lesions in HIV+ patients suggests that it may be useful to explore broader immunotherapy applications in this disease, potentially relevant in earlier stages of carcinogenesis.

### Supplementary information

ESM 1 (DOCX 18 kb)
