# Supplementary material for: Programmed death-ligand 1 (PD-L1) expression in cervical intraepithelial neoplasia and cervical squamous cell carcinoma of HIV-infected and non-infected patients
Source: Virchows Arch. 2023 Jun 21;484(3):507–16. doi: 10.1007/s00428-023-03580-z (PMC11021248; doi:10.1007/s00428-023-03580-z)
Supplement: Supplementary file 1 — (DOCX 18 kb) [file 428_2023_3580_MOESM1_ESM.docx]

Supplementary Tables

**Table S1a- Specimens (22C3 antibody)**

|  | **PD-L1 22C3 antibody** | | | | | |
| --- | --- | --- | --- | --- | --- | --- |
| Invasive SCC | **HIV+** | | | **HIV-** | | |
|  | Cases (no.) | Specimen | No. | Cases (no.) | Specimen | No. |
|  | 18 | Hysterectomy | 1 | 17 |  |  |
|  |  | Cone biopsy | 2 |  | Cone biopsy | 16 |
|  |  | Biopsy | 15 |  | Biopsy | 1 |

HIV, Human Immunodeficiency Virus; SCC, Squamous Cell Carcinoma; PD-L1- Programmed Death-Ligand 1; no, number

**Table S1b - Specimens (PD-L1 SP263 antibody)**

|  | SP263 PD-L1 Antibody | | | | |
| --- | --- | --- | --- | --- | --- |
|  | **HIV+** | | **HIV-** | | |
| Invasive SCC | No. of areas assessed | Specimen | | No. of areas assessed | Specimen |
|  | 13 areas (13 cases*) | Hysterectomy (n=1)  Biopsies (n=12) | | 17 areas  (17 cases**) | Cone (n=16)  Biopsy (n=1) |
| HSIL non-adjacent to SCC | 21 areas (21 cases) | Biopsies (n=21) | | 20 areas  (20 cases) | Biopsies (n=20) |
| HSIL adjacent to SCC | 8 areas (8 cases*) | Biopsies (n=7)  Hysterectomy (n=1) | | 9 areas  (9 cases**) | Cone (n=9) |
| LSIL | 20 areas (20 cases) | Biopsies (n=20) | | 20 areas  (20 cases) | Biopsies (n=20) |
| NILM | 20 areas (20 cases) | Biopsies (n=20) | | 20 areas  (20 cases) | Biopsies (n=20) |
| Total | 82 areas (74 cases) | Biopsies (n=73)  Hysterectomy (n=1) | | 77 areas  (77 cases) | Biopsies (n=21)  Cone (n=25) |
|  | Total number of specimens (n=151)  Total number of areas evaluated (n=159) | | | | |

* Same cases used to evaluate PD-L1 in invasive SSC areas and areas adjacent to SCC.

** Same cases used to evaluate PD-L1 in invasive SSC areas and areas adjacent to SCC.

HIV, Human Immunodeficiency Virus; NILM, Negative for Intraepithelial Lesion or Malignancy; LSIL, Low-Grade Squamous Intraepithelial Lesion; HSIL, High-Grade Squamous Intraepithelial Lesion; SCC, Squamous Cell Carcinoma; PD-L1- Programmed Death-Ligand 1*;* no-number
